# Anti-CASPR2 meningoencephalitis with thickened dura mater induced by various infections: A case report and literature review

**DOI:** 10.1097/MD.0000000000042873

**Published:** 2025-06-20

**Authors:** Pan Gao, Xiaowu Chen, Yuhao Li, Fangming Li, Hongya Zhang

**Affiliations:** a Department of Neurology, Shenzhen University General Hospital, Shenzhen, China; b The Education University of Hong Kong, Tai Po, New Territories, Hong Kong, China.

**Keywords:** case report, CASPR2, dura mater, infections, meningoencephalitis

## Abstract

**Rationale:**

With the update of novel autoantibodies and the expansion of the clinical spectrum, our understanding of autoimmune encephalitis (AE) is rapidly evolving. Anti-CASPR2 meningoencephalitis is a relatively rare condition that may be induced by infections.

**Patient concerns:**

A young man presented with 3 episodes of meningoencephalitis potentially triggered by possible viral, *Salmonella*, and severe acute respiratory syndrome coronavirus 2. Neuroimaging revealed a thickening of the cerebral dura mater. Laboratory tests found positive serum CASPR2 antibodies in the third episode.

**Diagnoses:**

Across all 3 episodes, similar clinical manifestations and imaging features were observed. Although autoantibodies in the previous 2 phases were negative, the possibility of infection-related anti-CASPR2 meningoencephalitis remains highly suspected.

**Interventions:**

The treatment regimen comprised antimicrobial agents, corticosteroid therapy, intravenous immunoglobulin, and rituximab administration.

**Outcomes:**

Following treatment, the patient’s condition improved with no recurrence to date. Repeat testing showed undetectable anti-CASPR2 immunoglobulin G in both serum and cerebrospinal fluid. Post-treatment contrast-enhanced magnetic resonance imaging demonstrated the resolution of dural thickening.

**Lessons:**

We further reviewed the mechanism of various infection-related AE and characteristics of CASPR2-related disease. To our knowledge, this is the first report of CASPR2 meningoencephalitis with thickened dura mater, indicating the importance of paying attention to antibody-negative AE and monitor antibodies repeatedly when necessary. In addition to immunotherapy, we recommend comprehensive management throughout the disease process.

## 1. Introduction

In recent years, antibody-positive autoimmune encephalitis (AE) has gradually gained public attention. With the update of novel autoantibodies and the expansion of the clinical spectrum, our understanding of AE is rapidly evolving. AE may be related to a variety of infections, including bacteria and viruses such as severe acute respiratory syndrome coronavirus 2 (SARS-CoV-2). Anti-CASPR2 meningoencephalitis is a relatively rare condition that can be similarly induced by infections. Different treatment strategies have been applied for CASPR2 encephalitis including steroids, intravenous immunoglobulin (IVIG), plasma exchange, azathioprine, cyclophosphamide, and/or rituximab. Herein, we detail a case of anti-CASPR2 meningoencephalitis with thickening of the cerebral dura mater, induced by several infections that have not previously been reported.

## 2. Case description

### 2.1. Phase I

**Figure 1.**
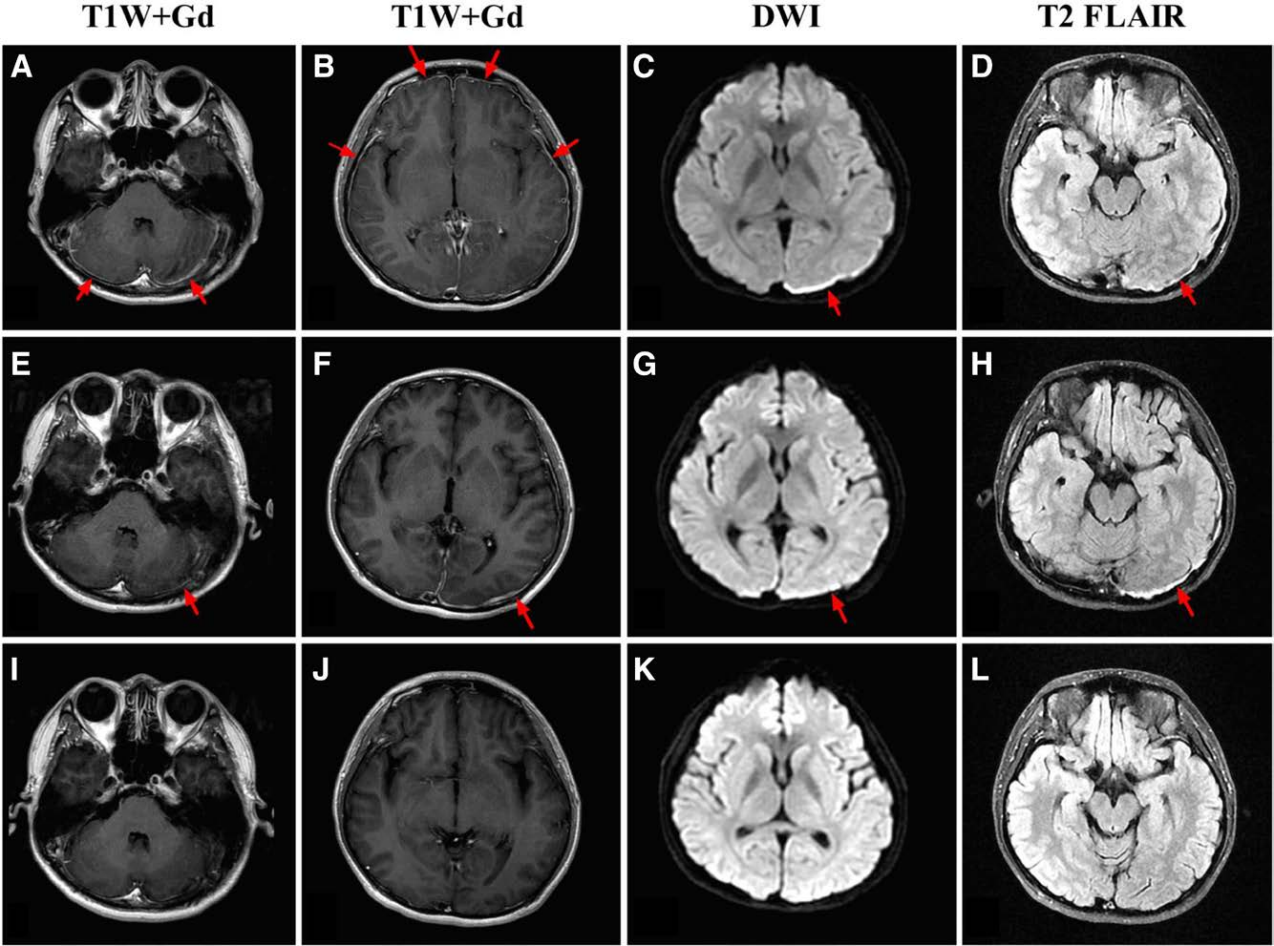
(A–D) In phase I, enhanced brain MRI revealed thickening of the bilateral dura mater, particularly on the left side. (E–H) In phase I, enhanced brain MRI revealed a slight improvement in the lesion of the dura mater during the process of immunotherapy. (I–L) In phase II, enhanced brain MRI revealed that thickening of dura mater was relieved after approximately 1 month of comprehensive treatment.

In mid-October 2020, an 18-year-old male presented with bilateral lower extremity muscle soreness following military training. Four days later, he developed psychiatric symptoms, including irrelevant answers, gibberish speech, irritability, and sleep disturbances. He denied any relevant medical, medication, epidemiological, or prodromal infection history, and further denied a history of tobacco and alcohol consumption. On admission, his body temperature was 37.5 °C, and his blood pressure, heart rate, and respiratory rate were normal. Neurological examination revealed a weakened tendon reflex in the right upper extremity and a probable positive Kernig sign. No other abnormalities were observed. Coronavirus disease-2019 (COVID-19) nucleic acid test results were negative. Routine blood tests, liver and renal function, electrolytes, procalcitonin level, C-reactive protein, erythrocyte sedimentation rate, and coagulation function were all within the normal ranges. Serum immunity indices revealed an increase in serum immunoglobulin G (IgG) at 32.2 g/L, whereas the remaining indices were normal, including immunoglobulin A, immunoglobulin M (IgM), complement C3, complement C4, IgG4, antinuclear antibody spectrum, cytoplasmic antineutrophil cytoplasmic antibody, perinuclear antineutrophil cytoplasmic antibody, myeloperoxidase, neutrophil proteinase 3, and rheumatoid factor. Tumour marker tests, including alpha-fetoprotein, carcinoma embryonic antigen, carcinoma antigen 125, carcinoma antigen 199, cytokeratin fragment 21-1, neuron-specific enolase, and prostate-specific antigen were within the normal ranges. Myocardial enzyme tests revealed an increased lactate dehydrogenase level of 5302 U/L, increased aspartate aminotransferase level of 843 U/L, increased creatine kinase level of 39,875 U/L, and increased myoglobin level of 684.6 ng/mL. A thyroid autoantibody test revealed an increase in thyroid globulin antibody of 98.92 IU/mL. The lymphocyte subset test revealed an increase in CD3‐/CD19+ (B cells) at 21.42% concurrent with a decrease in CD3‐/CD16+ 56 + Abs (js3/16 + 56) at 125.38 cells/μL. The cerebrospinal fluid (CSF) pressure was normal. Routine and biochemistry CSF tests were within normal ranges. Tests for the Japanese encephalitis antibody, Cryptococcus neoformans capsular antigen, and microbial DNA sequencing in CSF all also showed no abnormalities. Oligoclonal bands, AE antibodies (NMDAR, AMPAR1, AMPAR2, LGI1, CASPR2, and CABABR), and demyelinating antibodies (AQP4, MBP, MOG, and GFAP) in the serum and CSF were all negative. The results of electrocardiogram, chest CT, abdominal ultrasound, urinary ultrasound, and echocardiography results were normal. Brain magnetic resonance imaging (MRI) revealed bilateral thickening of the dura mater, particularly on the left side (Fig. 1A–D).

The results of evaluation were as follows: the patient presented with limbic encephalitis characterized by psychiatric symptoms, sleep disturbances, and a low fever, indicating an inflammatory response. Suspicious positive signs of meningeal irritation and thickening of the dura mater suggested meningeal involvement. Accordingly, the patient was diagnosed with autoimmune meningoencephalitis, potentially induced by a viral infection. Following admission, the patient was treated with acyclovir, olanzapine, phenobarbital, and venous fluid replacement. After the diagnosis was clarified, impulsion therapy of IVIG (0.4 g/kg/d*5d) and methylprednisolone (1g/d*3d, 500 mg/d*2d, 250 mg/d*1d) were administered. Reexamination of the brain MRI in early November 2020 revealed a slight improvement in the lesion of the dura mater during immunotherapy (Fig. 1E–H). The symptoms improved and he was discharged in mid-November 2020.

### 2.2. Phase II

Following discharge, the patient experienced a marked increase in fatigue, loss of emotions, and crying, accompanied by a low fever. The patient was subsequently readmitted to our hospital. The results of the COVID-19 nucleic acid test were still negative. Routine blood tests revealed an increase in white blood cell and neutrophil counts. Widal and Weil Felix tests revealed a positive Salmonella typhi O antibody titer of 1:160 and a positive Salmonella paratyphoid C antibody titer of 1:320. The serum galactomannan test revealed an increase in Aspergillus antigen at 0.61 (0.00–0.49) and the serum (1,3)-β-D-glucan test revealed an increase in fungal (1–3)-β-D-glucan at 210.4 pg/mL (<60 pg/mL). Quantitative determination of endotoxin levels and blood cultures revealed no significant abnormalities. The CSF pressure was normal. The CSF routine and biochemistry test, oligoclonal bands test, serum IgG4, serum and CSF AE antibodies (DPPX, IgLON5, GABAARα1, GABAARβ3, GlyRα1, mGluR5, D2R, Neurexin3α, GAD65), serum and CSF paraneoplastic syndrome antibodies (Hu, Yo, Ri, CV2, Ma2, Amphiphysin, Mal, SOX1, Tr/DNER, Zic4, GAD65, PKCγ, Recoverin, Titin), and CSF microbial RNA sequencing were all normal. Ultrasonography of the bilateral inguinal, neck, and axillary regions revealed bilateral cervical lymph node enlargement, indicating possible reactive hyperplasia, with no significant abnormal enlargement of the lymph nodes in the bilateral axilla and groin areas. Following lumbar puncture, the patient received IVIG (0.4 g/kg*5d) and methylprednisolone (60 mg/d). Considering the possibility of paratyphoid fever and fungal infection, levofloxacin and fluconazole were administered, while methylprednisolone was reduced to 30 mg/day for continued immune regulation treatment. In early December 2020, enhanced brain MRI revealed no bilateral thickening of the dura mater (Fig. 1I–L). Widal and Weil Felix tests revealed Salmonella typhi O antibody at a titer of 1:80 and Salmonella paratyphoid C antibody at a titer of 1:160. The patient’s condition subsequently improved, and he was discharged. However, the patient’s condition was not resolved.

### 2.3. Phase III

**Figure 2.**
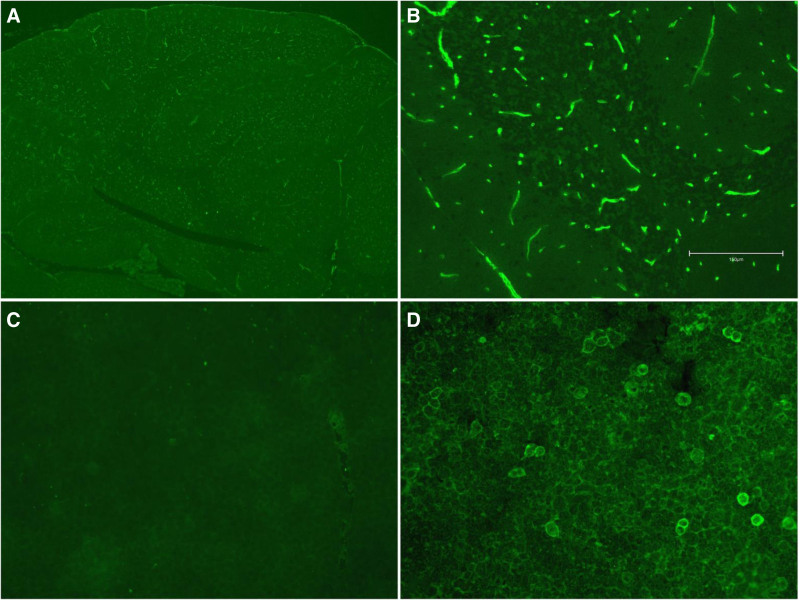
(A) The patient CSF immunofluorescence staining (TBA) at 4× magnification. (B) The patient CSF immunofluorescence staining (TBA) at 20× magnification showed vascular-like abnormal signal. (C) The patient CSF Caspr2 staining (CBA) was negative. (D) The patient serum Caspr2 staining (CBA) was positive at a dilution of 1:10.

**Figure 3.**
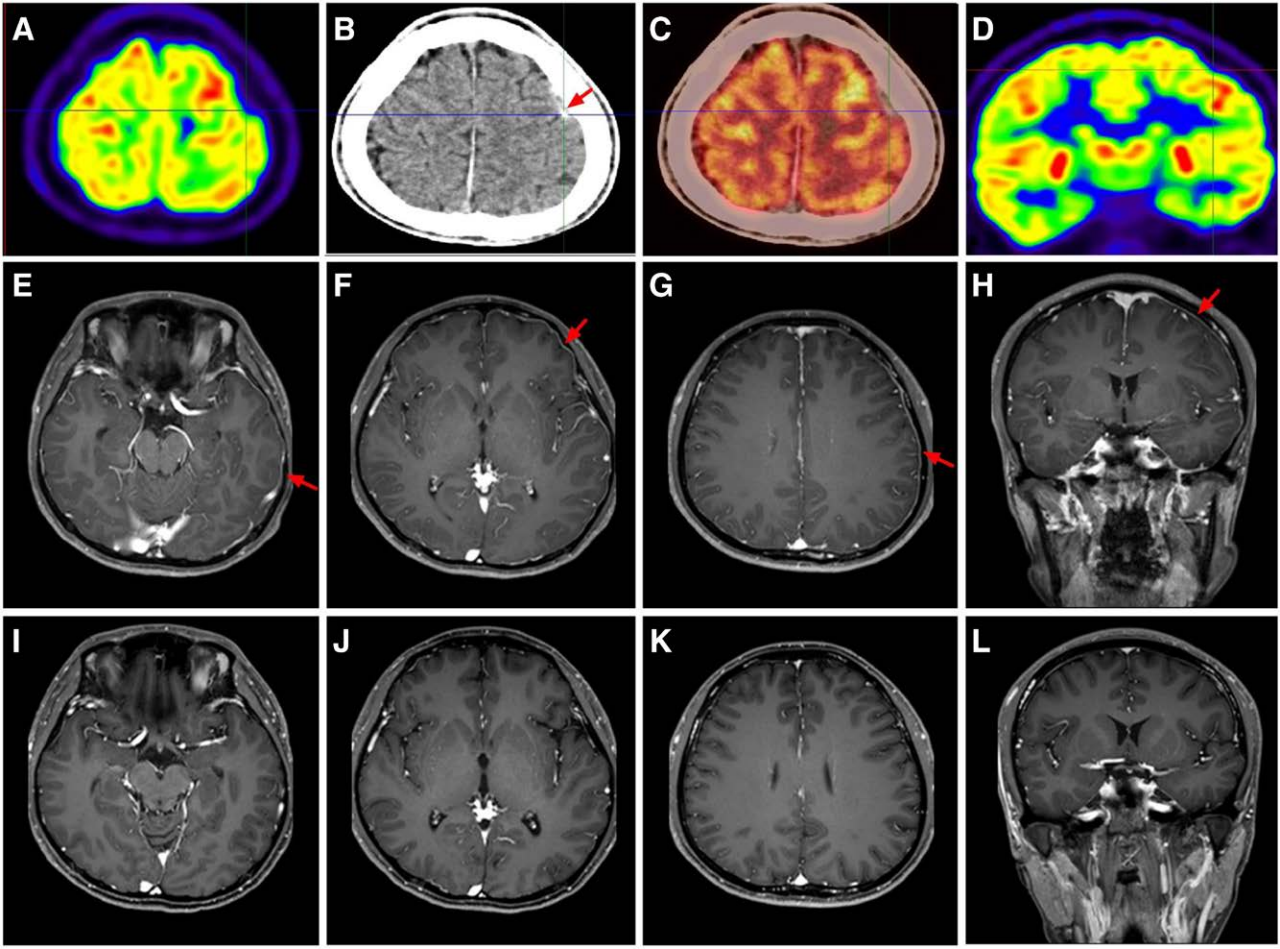
(A–D) In phase III, PET-CT of the brain revealed that the dura mater of the left frontal part was slightly thicker, metabolism was normal, and there were no obvious abnormalities in the brain parenchyma. (E–H) In phase III, the left dura mater was strengthened and thicker than the contralateral region in the enhanced brain MRI. (I–L) In phase III, the enhanced brain MRI showed that thickening of the dura mater was relieved after over 1 month of comprehensive treatment. PET-CT = positron emission tomography-computed tomography.

The patient again presented with a fever in late December 2022, with a maximum body temperature of 38 °C, and COVID-19 nucleic acid detection was positive. The patient ultimately developed sleep disorders 2 days later and psychiatric symptoms 6 days later. Physical examination revealed horizontal nystagmus to the left in both eyes with no other abnormalities. C-reactive protein and free thyroxine levels were slightly elevated. Serum cytokines tests further revealed an increase of interleukin (IL)-17 at 39.1 pg/mL (≤19 pg/mL). Routine and biochemical blood tests, Widal test, Weil–Felix test, and immunity indices (immunoglobulin A, IgG, IgM, complement C3, complement C4, IgG4, cytoplasmic antineutrophil cytoplasmic antibody, perinuclear antineutrophil cytoplasmic antibody, antinuclear antibody spectrum, myeloperoxidase, neutrophil proteinase 3, and anti-glomerular basement antibody) showed no other abnormal findings. CSF pressure, routine CSF tests, biochemical tests, CSF cryptococcal capsular antigen levels, and oligoclonal bands were all normal. On December 27, 2022 testing for serum and CSF AE antibodies (NMDAR, AMPAR1, AMPAR2, LGI1, CASPR2, CABABR, DPPX, IgLON5, GABAARα1, GABAARβ3, GlyRα1, mGluR5, D2R, Neurexin3α, GAD65) and ganglioside antibodies (GM1, GM2, GM3, GM4, GD1a, GD1b, GD2, GD3, GT1a, GT1b, GQ1b, sulfatide) were planned. All tests for CSF antibodies were negative. Serum anti-GT1a antibody IgG and serum anti-CASPR2 antibody IgG were positive at a titer of 1:10 (Fig. 2A–D). Thyroid ultrasound revealed an increased blood flow signal in the thyroid, cystic and solid nodules in the right lobe, classified according to the Chinese version of the thyroid imaging reporting and data system as category 3, with no surrounding enlarged lymph nodes. Enhanced brain MRI revealed that the left dura mater was strengthened and thicker than the contralateral dura (Fig. 3E–H). Positron emission tomography-computed tomography imaging of the brain showed that the dura mater of the left frontal part was slightly thicker, the metabolism was normal, and there was no obvious abnormality in the brain parenchyma (Fig. 3A–D).

The case summary was as follows: the patient had no manifestations of cranial nerve involvement or sensory disturbances, indicating that the anti-GT1a antibody was not responsible for the symptoms. Based on the above clinical manifestations, the positive serum CASPR2 antibodies, and thickening of the dura mater, the patient was diagnosed with CASPR2-related autoimmune encephalomeningitis. During his hospitalization, methylprednisolone (500 mg/d*3d) and IVIG (0.4 g/kg*5d) were administrated. Ganciclovir, levofloxacin, and ceftriaxone were further administered for anti-infection therapy. In early January 2023, rituximab (640 mg) was intravenously administered. The patient’s symptoms substantially improved. Reexamination of serum and CSF anti-CASPR2 antibody IgG levels were negative. Following discharge, the patient continued to receive oral methylprednisolone (40 mg/day) as maintenance therapy. Repeat brain MRI scan in February (Fig. 3I–L) revealed no obvious abnormalities.

**Figure 4.**
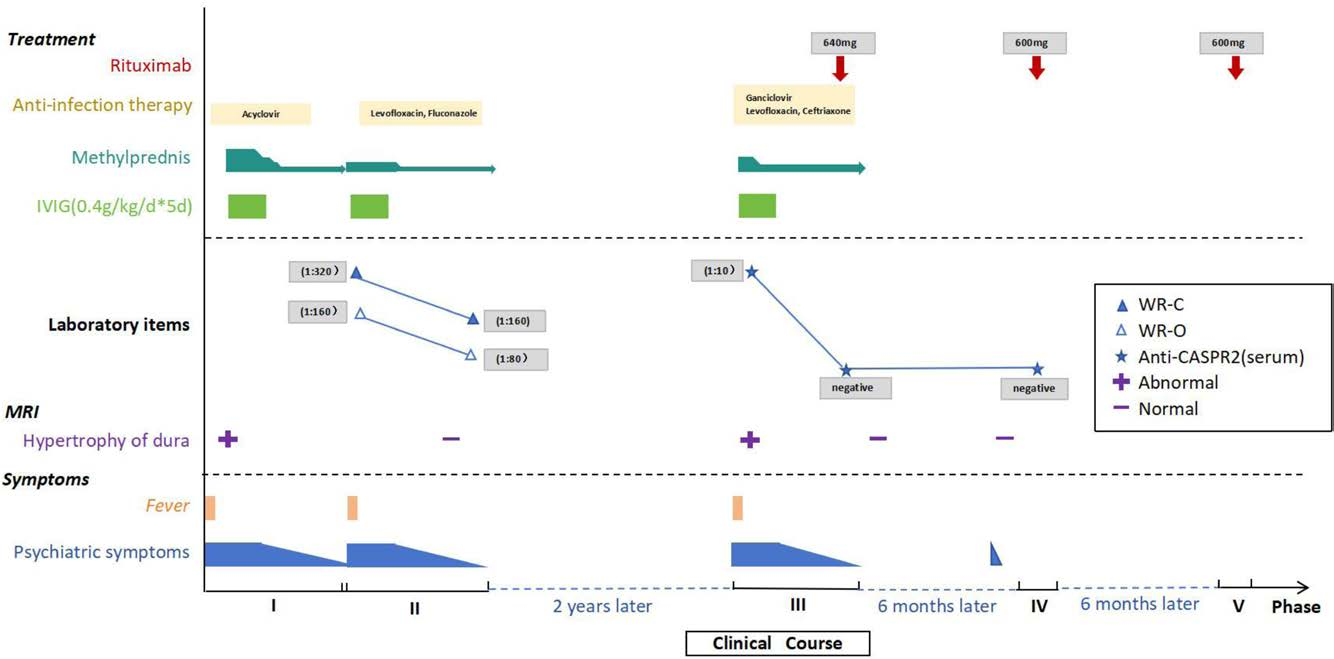
The clinical course of our case.

In June 2023, the young man again presented with obsessive-compulsive symptoms, such as repacking clothes for a short time after a long journey. During hospitalization in July 2023, routine CSF, biochemical, cytokine, oligoclonal bands, anti-CASPR2 antibody, both in the serum and CSF, were reexamined, and brain MRI was performed. No apparent abnormalities were observed. The patient was treated with intravenous rituximab (600 mg) therapy in July 2023 (Fig. 4, phase IV) and January 2024 (Fig. 4, phase V), as planned. To date, no recurrences have been reported.

## 3. Discussion and evaluation

As mentioned above, in phase I, the patient manifested with low fever, limbic encephalitis, probable meningeal irritation signs, and thickened dura mater. Antiviral therapies and immunotherapies were effective at this stage. Although the results of pathogenic assays and autoimmunity-related antibodies were negative, viral infection-induced autoimmune meningoencephalitis was diagnosed. However, the patient presented with a low fever and limbic encephalitis in phase II. Laboratory tests indicated paratyphoid fever and fungal infection, whereas autoimmunity-related antibodies in both serum and CSF were negative. The patient was diagnosed with Salmonella infection-induced AE. Following anti-infective therapy and immunotherapy, his symptoms improved, the titers of the Widal and Weil Felix tests decreased, and no thickening of the dura mater was observed. In phase III, the patient was readmitted with fever, SARS-CoV-2 infection, limbic encephalitis, and thickening of the left dura mater. The serum anti-CASPR2 antibody IgG test results were positive. The patient was subsequently diagnosed with CASPR2-related autoimmune meningoencephalitis. Reviewing the 3 phases, similar clinical manifestations and imaging characteristics were observed. Although autoantibodies in the previous 2 phases were negative, the possibility of infection-related anti-CASPR2 meningoencephalitis remains highly suspected. These 3 episodes of encephalitis were mediated by possible viral, Salmonella, and SARS-CoV-2 infection. This case indicates the importance of paying attention to antibody-negative AE and monitoring antibodies repeatedly when necessary, as the results of antibodies may be negative in the early stages.

The findings of previous studies have shown that the pathogenesis of AE may include disease triggers (infections), predisposing factors (human leukocyte antigen), and an interplay between these 2 factors.^[1]^ Various viral and bacterial infections can also be triggered. The herpes simplex virus (HSV) is the most frequently associated pathogen.^[2–4]^ Several possible mechanisms of post-viral AE have been proposed, as follows: 1. Direct invasion via the blood following disruption of the blood–brain barrier (BBB)^[2]^ during which anti-inflammatory molecules may enter the central nervous system (CNS) via the BBB.^[3]^ 2. Invasion of the olfactory tract. This hypothesis has been supported by a study showing that intranasal inoculation of HSV-1 in mice could induce NMDAR antibody production in over half of the tested animals.^[5]^ 3. Molecular mimicry,^[3]^ such as the similarity between NMDAR and HSV proteins. 4. Bystander effect.^[6]^ viruses may dissolve neurones, release antigens, and initiate autoimmune reactivity.^[1,7,8]^ 5. Relevant genetic deficiencies; in support of this hypothesis, one published study showed that 5% of patients with HSV encephalitis have a specific deficiency in the gene encoding toll-like receptor 3, which plays an important role in innate immune pathways, and 66% of patients with toll-like receptor 3 deficiency later develop AE.^[9]^ 6. Disruption of immune tolerance following recurrent infections.^[2]^ 7. Secondary encephalitis; in support of this, one study has shown that anti-CNS inflammation caused by NMDAR encephalitis may lead to latent viral DNA shedding or reactivation, resulting in secondary encephalitis.^[10]^

To date, no cases of AE induced by Salmonella or other enterobacteria have been reported. Noninfectious neurological complications of typhoid fever include encephalopathy, neuropsychiatric disorders, cerebral edema, cerebellar ataxia, Parkinsonism, acute disseminated encephalomyelitis, and brainstem encephalitis. Immune-mediated processes and endotoxaemia have been proposed as possible underlying mechanisms.^[11]^ The earlier case report of Salmonella Urbana encephalopathy revealed an increase in CSF cytokines (e.g., IL-6, IL-8) during the acute phase, and a decrease when the patient’s condition was improved, indicating that the “cytokine storm” may be one important mechanism.^[12]^ In regards to bacterial infection-related AE, another study of basal ganglia encephalitis triggered by repeated group A streptococcal infections suggested that Th17 lymphocytes may induce BBB leakage or microglia activation that would allow autoantibodies to enter the brain. Additionally, repeated untreated (or unresolved) infections can disrupt the immune tolerance to self-antigens, leading to the development of cross-reactive autoantibodies that target the CNS. Genetic susceptibility to autoimmunity may further play an important role in the pathogenesis.^[13]^ Overall, these hypotheses including “cytokine storm,” destruction of BBB, disruption of immune tolerance, and the genetic factors need further validation based on more cases and studies.

It has been clearly shown that SARS-CoV-2 mainly attacks the respiratory system, causing mild to severe respiratory diseases. However, this virus also affects the nervous system, and can potentially exhibit neurotropic and neuroinvasive properties.^[14,15]^ Published literature shows that approximately 36.4% of COVID-19 patients experience neurological symptoms, including AE,^[16]^ affecting both adults and children.^[17]^ A multicenter retrospective study reported that the incidence of encephalitis in COVID-19 patients with neurological symptoms is 2.2%.^[18]^ In a review of COVID-19-related AE, including 26 confirmed cases and 48 negative seroantibody cases, only 2 cases were positive for anti-CASPR2 antibody, which is relatively rare.^[19]^ Our patient was diagnosed with anti-CASPR2 meningoencephalitis, and may have been an asymptomatic SARS-CoV-2 carrier. A causal relationship can thus be inferred from the chronological order of the events. Autoimmune meningoencephalitis may be triggered by COVID-19. The pathophysiological mechanism of COVID-19-related AE remains unclear; however, 4 possible mechanisms have so far been proposed. The first possible mechanism is molecular mimicry between the COVID-19 virus protein and neural autoantigens, leading to intrathecal or systemic autoantibody synthesis in the host, recognition of autoantigens as foreign antigens, and cross-reaction with them, resulting in damage to many systems, including the central nervous system.^[6,20,21]^ The second possible mechanism is the induction of a “cytokine storm.” In this process, the host immune system is overactivated in response to COVID-19, resulting in the production of large amounts of inflammatory cytokines (e.g., IL-6) that migrate to the central nervous system, causing encephalitis.^[21,22]^ The third possible mechanism is invasion via the blood after the virus disrupts the blood–brain barrier via SARS-CoV-2-ACE2 receptor-mediated vascular damage.^[23–25]^ The fourth possible mechanism is that SARS-CoV-2 directly invades the central nervous system through the cribriform plate, olfactory bulb, or other cranial nerves, which has been supported by the involvement of these systems in COVID-19.^[26]^

CASPR2 is a cell adhesion molecule belonging to the neurexin IV superfamily. CASPR2 is encoded by the contactin-associated protein-like 2 gene on chromosome 7q35.^[27]^ In both the central and peripheral nervous systems, transmembrane axonal complexes formed by CASPR2 and contactin-2 (also known as [transient axonal glycoprotein] TAG-1) are adjacent to the axonal membranes of VGKC. CASPR2 aggregates near voltage gated potassium 1 channels in the paranodal region, regulating the formation of different axonal domains around the Ranvier node, and stabilizing Ranvier node conduction, thus regulating axonal excitability.^[28,29]^ CASPR2 is widely expressed in the inhibitory neurones of the CNS, where it connects to contact-2 before synapses and gephyrin after synapses, and may play a crucial role in the formation of synaptic networks as a cell recognition molecule.^[30]^ Titulae et al demonstrated that the CASPR2 antibody may exert its pathogenicity by blocking the function of CASPR2 or inhibiting protein–protein interactions, rather than through internalization or complement-mediated toxicity.^[31]^ In reported cases, the detection rate of antibodies in the serum was much higher than that in the cerebrospinal fluid. This may be because the peripheral immune system is activated before the synthesis of intrathecal autoantibodies, although CSF anti-CASPR2 antibodies may further be detected in subsequent longitudinal monitoring.^[32]^ Currently, all IgG subtypes (IgG1–4) have been reported in CASPR2-related diseases, among which IgG1 and IgG4 are the most common.^[33]^ Two retrospective studies have previously suggested a similar ratio of serum IgG1 to IgG4; however, IgG4 was more common in the CSF than IgG1.^[33,34]^ The IgG4 subtype of anti-CASPR2 antibodies may play a fundamental role in the pathogenic process by directly acting on CASPR2, while complement-mediated cell death induced by the IgG1 subtype may also be involved.^[34]^

CASPR2 is located in the limbic system, basal ganglia, neurones of other motor areas, and sensory pathways, and is particularly abundant in the temporal lobe. The most common MRI findings have been found to be T2 hyperintensities in the medial temporal lobe, followed by hippocampal atrophy, middle temporal gyrus sclerosis, and hippocampal sclerosis. The most common FDG-PET finding is hypometabolism in the temporal lobe.^[35]^ Abnormalities have also been reported in the frontal lobe, parietal lobe, occipital lobe, brainstem, cerebellum, basal ganglia, insula.^[32,35–37]^ and frontal cortical and subcortical white matter.^[38]^ The manifestations of CASPR2 encephalitis are diverse, including limbic encephalitis, Morvan syndrome, and peripheral nerve hyperexcitability, which are rare in other encephalitis.^[36,39]^ In recent years, the clinical spectrum of CASPR2-related diseases has become more diverse, with new manifestations including Parkinson disease,^[40]^ non-paraneoplastic cerebellar ataxia,^[35,41,42]^ Guillain-Barre syndrome,^[43]^ chorea,^[44]^ neuropathic pain,^[45]^ Creutzfeldt-Jakob disease,^[46]^ amyotrophic lateral sclerosis with frontotemporal dementia syndrome,^[47]^ orthostatic myoclonus,^[48]^ and eyelid tremor.^[49]^ The majority of CASPR2 patients are male, with a median age of 54 years; however, rare pediatric cases have also been reported, with a median duration of approximately 12 months. CASPR2-related diseases may occur comorbid with Myasthenia gravis, Guillain-Barré syndrome, chronic inflammatory demyelinating polyneuropathy, neuromyelitis optica spectrum disorders, multiple sclerosis and myelitis.^[35]^ Approximately 20% of patients have thymomas, most commonly those with Morvan syndrome and neuromyotonia.^[45]^ CASPR2-related diseases are also associated with neurodevelopmental disorders such as autism, mental retardation, and epilepsy.^[39,50,51]^ Our patient was a young man who presented with various infections, meningeal involvement and limbic encephalitis. This is the first report of CASPR2 meningoencephalitis involving thickening of the dura mater, reminding clinicians that the possibility of CASPR2 meningoencephalitis should also be considered when imaging only reveals thickening of the dura mater. However, the mechanisms underlying the involvement of the dura mater remain unknown, and need to be explored in animal model experiments and pathological studies.

Based on the consensus regarding the diagnosis and treatment of AE,^[52,53]^ a variety of immunotherapies have been applied to treat CASPR2 encephalitis; however, there have been no head-to-head studies on the different treatment strategies. One retrospective analysis included treatment methods for steroids, IVIG, plasma exchange, azathioprine, cyclophosphamide, and/or rituximab. In their patient cohort, 39% fully recovered, 52% were partially relieved, and 25% had clinical recurrence.^[45]^ One potential strategy for treating this disease is to reduce the number of B cells that produce IgG4. Rituximab, a monoclonal antibody against CD20, responds well to CASPR2 encephalitis.^[54,55]^ However, strategies targeting other components of the immune system, such as inhibiting the complement or T cells, may be ineffective.^[56]^ Our patient’s symptoms improved following treatment with steroids, immunoglobulins, and rituximab, and there has been no recurrence to date, which is consistent with the mainstream treatment regimens for CASPR2 encephalitis.

## 4. Conclusions

This is the first report of CASPR2 meningoencephalitis with thickened dura mater which was mediated by possible viral, Salmonella, and SARS-CoV-2 infection, reminding clinicians that the possibility of CASPR2 meningoencephalitis should also be considered when imaging only reveals thickening of the dura mater, also indicating the importance of paying attention to antibody-negative AE and monitoring antibodies repeatedly when necessary when the results of antibodies may be negative in the early stages. In addition to immunotherapy, one of the crucial measures for managing CASPR2-related AE or meningoencephalitis is the prevention of infections caused by viral, bacterial, and other rare pathogens. During immunosuppressive therapy to prevent disease recurrence and progression, some patients may develop opportunistic infections that can worsen their condition. We recommend comprehensive management throughout the disease process, dynamic monitoring of immune indicators such as IgG, IgM, and B lymphocytes, and advising patients to maintain a regular schedule and appropriate exercise, all of which may improve the disease.

## Author contributions

**Conceptualization:** Hongya Zhang.

**Methodology:** Fangming Li.

**Resources:** Xiaowu Chen.

**Supervision:** Fangming Li.

**Visualization:** Yuhao Li, Pan Gao.

**Writing – original draft:** Pan Gao.

**Writing – review & editing:** Hongya Zhang, Fangming Li.
